# Evidence of *Helicobacter* spp. in Saliva and Gastric Mucosa of Domestic Dogs in the Central Region of Rio Grande do Sul, Brazil

**DOI:** 10.1155/2021/8857231

**Published:** 2021-01-28

**Authors:** Daniel D. Guerra Segundo, Camila B. E. Mello, Juliana F. Cargnelutti, Mariana M. Flores, Luís F. Pedrotti, Bernardo N. Antunes, Vanessa Milech, Omar G. Velasquez, Letícia R. Martins, Saulo T. L. Pinto Filho

**Affiliations:** ^1^Programa de Pós-Graduação Em Medicina Veterinária, Universidade Federal de Santa Maria, Santa Maria 97105-900, Brazil; ^2^Departamento de Medicina Veterinária Preventiva, Universidade Federal de Santa Maria, Santa Maria 97105-900, Brazil; ^3^Departamento de Patologia, Universidade Federal de Santa Maria, Santa Maria 97105-900, Brazil; ^4^Departamento de Clínica de Pequenos Animais, Universidade Federal de Santa Maria, Santa Maria 97105-900, Brazil

## Abstract

*Helicobacter pylori* is a spiral-shaped bacterium, which plays a role in the aetiology of gastric diseases in humans. Non-*H*. *pylori Helicobacter* (NHPH) species naturally colonise the stomach of animals and also induce gastric lesions in humans, highlighting their zoonotic importance. We evaluated the gastric bacterial colonisation density and gastric lesions and sought to identify the main phylogenetic groups of the Helicobacter spp. obtained from dogs in the central region of Rio Grande do Sul, Brazil, with this study aiming to investigate the occurrence of *Helicobacter* spp. in saliva and gastric samples from these dogs. This study included 35 dogs and used analysis such as cytology, histopathology, PCR, rapid urease testing, and phylogenetic analysis. Of the dogs, 94.3% were positive for *Helicobacter* spp., and these bacteria were present in the stomach of 32 dogs and saliva of eight. Respectively, eight, 15, and nine dogs had mild, moderate, and severe colonisation. Lymphocytic-plasmacytic infiltrate was the main gastric lesion. However, the presence of *Helicobacter* and the density appeared to be unrelated to the gastric lesions. The samples possessed a high nucleotide identity with remarkably similar sequences among some of the species of NHPH such as *H*. *heilmannii s.s.*, *H*. *salomonis*, *H. felis,* and *H. bizzozeronii.* The saliva of domestic dogs, even of those who appear clinically healthy, can cause *Helicobacter* infection in humans and other animals, with, in these dogs, increased density, occurrence rate, and predominance of NHPH of zoonotic importance being found in the stomach with a lower occurrence of *Helicobacter* spp. in the saliva.

## 1. Introduction

*Helicobacter* spp. are spiral-shaped mobile Gram-negative bacteria with tropism for the gastric mucosa of humans and animals [1]. *Helicobacter pylori* (*H. pylori)* was the first species isolated from the *Helicobacter* genus by Australian researchers Marshall and Warren in 1983 [2]. Further research has shown that humans are the natural host of *H*. *pylori* and have established this species as the primary aetiology of peptic ulcers and gastric neoplasms [3]. Years later, in samples of gastric mucosa from humans, another spiral-shaped bacteria with similar morphological features to *H*. *pylori* was documented, and this microbe was found to be remarkably similar to the spiral-shaped bacteria found in the gastric mucosa of domestic animals [4]. This new spiral bacteria was later classified by Solnick et al. [5] as *Helicobacter heilmannii*. However, phylogenetic studies were later employed utilising the 16 rRNA target gene, and these studies found instead that this bacteria belonged to a range of several *Helicobacter* species isolated from domestic and wild animals, such as *H*. *felis*, *H*. *bizzozeronii*, *H*. *salomonis*, *Helicobacter heilmannii sensu stricto* (*H*. *heilmannii s.s.*), *H*. *bílis*, *H. cynogastricus*, and *H. baculiformis* [6]. Therefore, in order to organise this large group of bacteria into a single term, they were consensually denominated as *non-Helicobacter pylori Helicobacter* (NHPH) [7].

NHPH are the target of various studies due to their relationship with upper digestive tract illnesses in humans and their zoonotic importance [8]. Dogs are the natural hosts of NHPH and harbour this bacteria in their gastric mucosa, gut, and oral cavity; thus, gastric juice, saliva, and faeces are possible sources of transmission for this bacteria to infect humans [9–11]. In dogs, the main species of NHPH found are *H. heilmannii s.s*, *H. bizzozeronii*, *H. salomonis*, *H. felis*, *and H. canis* [9, 11].

Chronic inflammation of the gastric mucosal tissue, peptic ulcers, and gastric mucosa associated lymphoid tissue lymphoma are the clinical alterations described in humans with NHPH infection [12, 13]. In human populations, NHPH has a prevalence of 0.5% in developed countries [14] and 6.2%–15% in underdeveloped countries [15, 16]. Thus, countries with lower socioeconomic development tend to have a higher prevalence of infected people with NHPH [17]. However, information regarding the importance of domestics dogs as reservoirs for this bacteria and the data related to the number of NHPH occurrences in the canine population of these countries has not yet been explicated [18].

Due to the zoonotic implications of NHPH, compounded by the high density of domestic animals, and sanitary problems affecting Brazil, it is important to elucidate this information to assist with future studies of public health. Moreover, the geographic variation could affect the prevalence of the *Helicobacter* species [11, 18]. Thus, the aim of this study was to investigate the presence of *Helicobacter* spp. from saliva and the gastric mucosa of domestic dogs. Moreover, this study documented the gastric bacterial colonisation density, the number of gastric lesions present and sought to identify the main phylogenetic groups of *Helicobacter* spp. found in dogs from the central region of Rio Grande do Sul.

## 2. Materials and Methods

### 2.1. Ethics Statement

All the procedures were performed with the consent of owners of the dogs and the study protocol was approved by the Animal Ethics Committee of Universidade Federal de Santa Maria (Approval number 2827081018) and conducted in accordance with national guidelines and regulations for the care and use of laboratory animals established by the National Council for Animal Experimentation Control (CONCEA), Brazil.

### 2.2. Animals

This study collected saliva and gastric mucosal samples from 35 client-owned domestic dogs. They included seven males and 28 females, ranging from seven months to 14 years of age. Twenty-three of these dogs were clinically normal (all females) and in these animals selected, any alteration or underlying disease were discarded through the history, physical examination, complete blood count, serum biochemistry profile (creatinine, blood urea nitrogen, alkaline phosphatase, albumin, and alanine aminotransaminase). In addition, these healthy animals were submitted to sterilization procedure in the counterpart of the upper digestive endoscopy and gastric biopsy sample collection. Twelve had a history of chronic vomiting (seven males and five females; seven dogs with chronic vomiting only, three with chronic vomiting accompanied with weight loss and alteration in appetite, one with chronic vomiting accompanied with diarrhea, and one with chronic vomiting accompanied with hematemesis). These symptomatic animals were only submitted to upper digestive endoscopy and gastric biopsy sample collection due to gastric symptoms. For selection of the dogs classified with chronic vomiting (related to chronic gastritis) in this study, the inclusion criteria employed was the confirmation of a history of clinical signs such as vomiting episodes accompanied or not with diarrhea, weight loss, and alteration in appetite, for a period superior to 3 weeks [19, 20]. Common differential diagnostics of chronic vomiting, such as drugs toxicity, foreign bodies, hepatic failure, or renal disease, were discarded by history, physical examination, complete blood count, serum biochemistry profile (creatinine, blood urea nitrogen, alkaline phosphatase, albumin and alanine aminotransaminase) and ultrasonography and/or radiography previous to procedure and by upper digestive endoscopy on the moment of study. These animals were arising from the municipalities of Santa Maria and Itaara, located in the central region of the State of the Rio Grande do Sul, Brazil. Preceding sample collection, the dogs did not receive effective eradication protocols for gastric *Helicobacter* spp. employing the use of the combination of amoxicillin and clarithromycin or metronidazole and proton pump inhibitor (omeprazole or lansoprazole) or famotidine for 14 days [21, 22]. However, antibiotics such as ampicillin, amikacin, metronidazole, and sulfamethoxazole-trimethoprim were used per three days in four dogs with clinical signs (dogs number: n.2 has received ampicillin and metronidazole; n.4 has received sulfamethoxazole and trimethoprim; n.21 has received metronidazole; and n.27 has received ampicillin, amikacin, and metronidazole) prior to sample collections.

### 2.3. Sample Collection

Following a fasting period of 12 hours, all dogs were anesthetically induced with propofol (4 mg/kg) and held in an anaesthetised state with isoflurane and 100% oxygen. Saliva collection was performed with a sterile swab prior to the endoscopic procedure (to prevent cross-contamination of saliva samples with gastric secretions), and the samples were stored in sterile normal saline and frozen at −80°C until further processing. The collection of gastric biopsies was performed with a flexible endoscope (Karl Storz GmbH & Co.KG., Tuttlingen, Germany) and 2.3 mm diameter biopsy forceps (Changzhou Jiuhong Medical Instrument Co. Ltd., Changzhou, China). Following the mucosal evaluation, four biopsy samples were collected from the body and gastric antrum. Impression smears of the two gastric biopsy samples from the body and antrum were prepared on an air-dried slide. Then impression smears from the same samples were placed into two tubes (one tube for each gastric zone) containing 10% formalin until further processing. One sample of each gastric zone was submitted for the rapid urease test. For molecular analysis, one sample from each gastric zone was stored in a tube containing sterile normal saline and frozen at −80°C until further processing.

In procedures using additional analgesic drugs and loco-regional anesthesia, sterilization in those asymptomatic female dogs was performed postgastroscopy employing the conventional open surgical spaying or laparoscopic-assisted method.

### 2.4. Rapid Urease Test (RUT)

RUT was employed with a Urease test kit (RenyLab, Barbacena, Brazil). Following collection, the biopsy samples for each gastric zone were placed together into a test tube and evaluated for 60 minutes. Colour transformation from yellow to pink within 60 minutes was considered positive, while, no colour transformation within 60 minutes was considered negative.

### 2.5. Cytology

Following the impression smears of the biopsy samples, the slides corresponding to both gastric zones were stained with a quick panoptic stain. The gastric bacterial colonisation densities (the mean of 10 microscopic fields at ×400) were recorded as follows (−0) no bacteria; (+1 = mild) less than 10 bacteria per field; (+2 = moderate) 10–50 bacteria per field, and (+3 = severe) more than 50 bacteria per field [23, 24].

### 2.6. Histopathology

The formalin-fixed gastric samples for the body and the antrum were sectioned and stained with haematoxylin and eosin and then processed routinely. The gastric samples were evaluated by using the World Small Animal Veterinary Association (WSAVA) gastrointestinal standardisation visual analogue scale [25], where the attributed scores are based on the gastric lesion severity and recorded as follows: (0) lesion not observed; (1) mild lesion; (2) moderate lesion; and (3) severe lesion. The values obtained in the evaluation of the three random fields for each gastric zone were calculated and expressed with the mean of the gastritis severity score.

### 2.7. DNA Extraction and PCR Amplification

Some animal samples, such as faeces and saliva, had PCR inhibitors. To minimize this issue, we used DNA extraction and PCR protocol used worldwide in the DNA microorganism detection. Thus, DNA was extracted from the saliva and gastric biopsies with the DNeasy Blood and Tissue Kit (Qiagen N.V., Hilden, Germany), following the manufacturer's instructions. Total DNA was extracted for each sample and submitted to PCR testing, which used the following oligonucleotide primers (F-5′- AAC GAT GAA GCT TCT AGC TTG CTA-3′; R-5′- GTG CTT ATT CST NAG ATA CCG TCA T-3′), which amplified a fragment of 399 base pairs (bp) for the 16S rRNA gene of the *Helicobacter* spp. [26]. PCR testing involved 1x buffer of PCR containing MgCl2, 10 mM of dNTPs (0,2 mM of each), 10 pmol of each primer, 1 U of Taq polymerase enzyme (Promega, Madison, Wisconsin, USA), and qsp water; following these conditions, the initial denaturation was 95°C per 5 minutes, followed by 32 cycles at 94°C–30 s; 62°C–30 s to annealing of the initiators and 72°C–30 s for chain extension; and final extension for 3 minutes at 72°C. The amplified product was analysed by electrophoresis in an agarose gel 1% (60V, 1 hour and 30 minutes), using GelRed® (Biotium, California, USA) and visualised by a transilluminator with ultraviolet light. In all of the amplifications, the positive control was obtained from the gastric mucosal samples from a dog known positive for *Helicobacter* spp. and the negative controls were ultrapure water samples.

### 2.8. Sequencing and Phylogenetic Analysis

The amplification products for PCR were submitted in duplicate for nucleotide sequencing via the Sanger method using Prism 3500 Genetic Analyzer equipment (Life Technologies, California, USA). The consensus sequence from the Staden Package program was used to start the nucleotide sequences [27]. Phylogenetic analysis utilised the consensus sequence for each of the amplified samples and the nucleotide sequences of the *Helicobacter* spp. obtained from the GenBank database (http://www.ncbi.nlm.nih.gov). The sequences were edited and aligned using the BioEdit Alignment Editor software suite, version 7.0.5.3 [28]. The phylogenetic tree was constructed using the MEGA X software [29].

### 2.9. Criteria for Positive Dogs with *Helicobacter* spp

A dog was considered positive for *Helicobacter* spp. when there was a positive result at least one test (cytology; RUT; or 16S rRNA PCR assay).

### 2.10. Statistical Analyses

Statistical analyses were then performed using the statistical software Action Stat Pro (Estatcamp, São Carlos, Brazil). Kruskal–Wallis test was employed to compare the bacterial colonisation density scores between the regions of the body and antrum. The Spearman test was used to evaluate the correlation between the scores of the histologic gastric lesions and the gastric bacterial colonisation density scores in both of the gastric zones. Fisher's exact test was used to determine if the *Helicobacter* infection was associated with gastric lesions in the histopathology findings. The significance level used was *P* < 0.05 for all statistic evaluations.

## 3. Results

### 3.1. Detection of *Helicobacter* spp. and Gastric Bacterial Colonisation Density (Table 1)

In accordance with RUT and cytology, 88.5% (31/35) and 91.4% (32/35) of the dogs were positive, respectively. The spiral-shaped bacteria observed by cytology on all samples resembled NHPH morphology. Detection of the 16S rRNA, specific to the *Helicobacter* genus, was positive 25.7% in saliva (9/35) and 74.2% (26/35) in the gastric mucosa.

**Figure 1:**
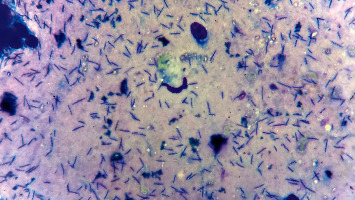
Impression smear of the gastric biopsy sample showing the presence of numerous *Helicobacter* organisms (Quick panoptic stain, ×1000).

Through the evaluation of gastric bacterial colonisation density scores (Figure 1), 22.8% (8/35) of the dogs showed a mild score, 42.8% (15/35) a moderate score, and 25.7% (9/35) a severe score. In dogs with chronic vomiting, the gastric bacterial colonisation density was 25% mild score (3/12) and 50% moderate score (6/12), but in 25% of the dogs, the bacteria was not present (3/12). All asymptomatic dogs were positive for the presence of *Helicobacter* spp.; 21.8% of the dogs showed a mild score (5/23), 39.1% moderate score (9/23), and 39.1% a severe score (9/23). There were no statistically significant differences in comparison with scores between the body and the antrum (*P*=0.8).

### 3.2. Findings of the Upper Digestive Endoscopy and Histopathology

**Figure 2:**
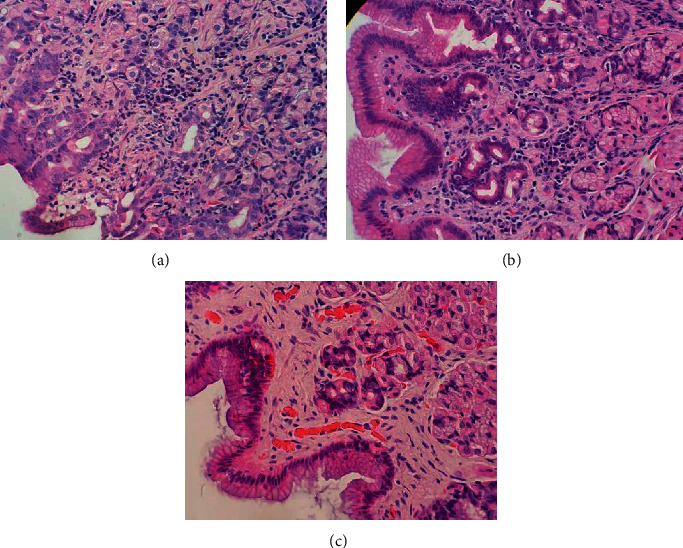
Gastric mucosa (body, A and B; antrum, C) biopsy sections. (a) Lamina propia with a high quantity of polymorphonuclear cells (asterisk), mainly eosinophilic infiltrate. (b) Lamina propia showed a high quantity of mononuclear cells (asterisk), with a predominance of plasmocytes. (c) Image of the normal gastric mucosa. (Haematoxylin and eosin stain, ×400).

Endoscopic evaluation of three dogs without *Helicobacter* gastric presence showed macroscopic alterations, such as patchy erythema (2/3), spotty erythema (1/3), oedema (1/3), superficial irregularities (1/3), and vascular pattern visibility (1/3). Moreover, 32 dogs with *Helicobacter* gastric presence showed the following macroscopic alterations observed patchy erythema (3/32), spotty erythema (10/32), linear erythema (1/32), oedema (3/32), and vascular pattern visibility (3/32). Based on the WSAVA standards, for the three dogs without *Helicobacter* gastric presence, only one showed gastric lesions, while the 32 dogs with *Helicobacter* gastric presence, 21 showed gastric lesions, 20 mild gastritis, and one moderate gastritis (Table 2). The gastric lesion patterns found in the 35 dogs were lymphocytic-plasmacytic infiltrates (18/35), neutrophilic infiltrates (9/35), eosinophilic infiltrates (9/35), intraepithelial lymphocytes (4/35), fibrosis, and mucosal atrophy (3/35), and gastric lymphofollicular hyperplasia (3/35) (Tables 2 and 3) (Figure 2).

Although the number of lymphocytic-plasmacytic infiltrates was high in the animals with *Helicobacter* gastric presence (Table 2); statistically, this was not associated with a known pattern of gastric lesions (*P* > 0.05). In addition, there was no correlation between the scores of histologic lesions with gastric bacterial colonisation density scores in the body (*P*=0.3) and antrum (*P*=0.9).

### 3.3. Nucleotide Identity and Phylogenetic Analysis

**Figure 3:**
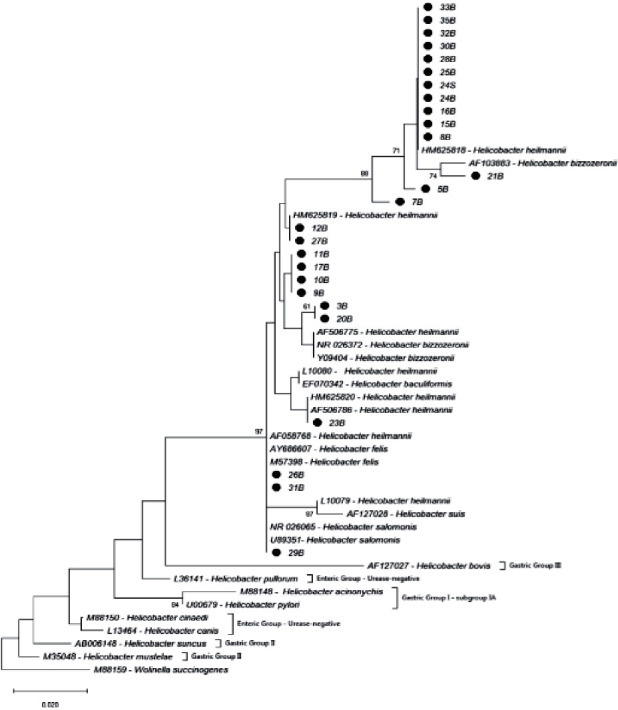
Phylogenetic tree based in the nucleotide sequence of 16S rRNA gene of *Helicobacter* spp. The tree was build employing the Neighbor-Joining method with a *bootstrap* of 1000 replicates. The evolutionary distances were computed using of Kimura-2 method. Values above 60% are showed. The sequences obtained of this study are highlighted with a black circle; n°B, animal number/gastric biopsy sample; n°S, animal number/saliva sample.

The identity of the nucleotides between the sequences obtained varied by 94.3 to 100%. The identity was high too when these sequences were compared with sequences of *H. heilmannii* (HM625818; 95.5 to 100%), *H. salomonis* (U89351; 94.3 to 100%), *H. felis* (AY686607; 94.3 to 100%), and *H. bizzozeronii* (NR026372; 94.9 to 99.3%). The identity of the nucleotide changed from 89.1% to 94.2% when compared with the sequences of *H. pylori* (U00679) samples (Figure 3).

## 4. Discussion

The occurrence of *Helicobacter* spp. (94.3%) in the dogs belonging to the Brazilian geographic region of this study was higher than that of the dogs from Japan (34.7%) [22], South Korea (78.4%) [30], Denmark (76.7%) [31], Germany (82%) [32], and Portugal (87%) [33]. However, our results were similar with dogs of Poland (96.7%) [11], Iran (95%) [34], Venezuela (95%) [18], and Costa Rica (95%) [35]. Interestingly, these results were different from those observed in human populations, whose occurrence of *Helicobacter* spp. increased according to the socioeconomic underdevelopment of the region [17], with the aforementioned results demonstrating the high level of geographic variation for these bacteria in domestic dog populations, regardless of socioeconomic level. However, additional data from developed and underdeveloped countries are required in order to correlate the *Helicobacter* spp. prevalence in populations of domestic dogs from different geographic regions [18, 33].

Through the evaluation of gastric bacterial colonisation density scores, 22.8% (8/35) of the dogs showed a mild score, 42.8% (15/35) a moderate score, and 25.7% (9/35) a severe score. Thus, more than half of the subject dogs were reservoirs of a substantial gastric colonisation density for NHPH.

During the gastroscopy, the main alteration observed was erythema; however, none of the dogs showed the presence of erosions or ulcerations that were described by Kubota-Aizawa et al. [22] and Suárez-Esquivel et al. [35]. Furthermore, some of the gastroscopy findings failed to match the histological findings. For instance, when microscopic lesions were observed, the macroscopic evaluation did not always exhibit alterations; however, these findings are not abnormal [19]. Therefore, the diagnosis of gastritis should not be based solely upon gastroscopy visualisation, as biopsy collection is indispensable to confirm the diagnosis of gastritis [36].

The main histologic changes observed in the dogs of this study were the presence of lymphocytic-plasmacytic infiltrates, which were commonly observed when NHPH was present [33]. Nevertheless, NHPH factors regarding the gastric presence and gastric bacterial colonisation density were not found to be correlated with the gastric lesions and severity scores, and this result was similar to other authors' results [9, 18]. Moreover, the virulence and pathogenicity could vary between the different *Helicobacter* species and strains within an NHPH infection [37]. Kubota-Aizawa et al. [22] suggest *in vitro* cultivation of each NHPH species and experimental infection of canine gastric cell lines or dogs to investigate the pathogenicity of each NHPH species.

This study was the first conducted in Brazil and investigated the presence of *Helicobacter* in the saliva of dogs. The saliva may exert an important role transmission source of NHPH to humans and other domestic animals [11]. In addition, some authors have suggested that the oral cavity could be a potential niche of *Helicobacter* spp. colonisation [10]. In this study, the results revealed divergent findings from those of other studies. Commonly, a high prevalence of 71.1% to 100% of *Helicobacter* spp. was detected in the saliva of canine populations and documented [9, 10]; however, the present study observed an occurrence significantly lower, at 25.7% (9/35). Another novel finding was that one dog was positive for NHPH in the saliva and negative in the gastric mucosa, which has not been reported by another study in a canine population. This observation has been previously described in cats [24]. However, we cannot affirm this based only on a case, but two hypotheses arose with this finding: It is possible that dogs with a gastric absence of *Helicobacter* spp. can harbour this bacteria in the oral cavity; or, this finding could be resultant of the very low number of *Helicobacters* distributed in the gastric mucosa of this dog, resulting in the absence of positivity by different methods of detection in the samples evaluated (false negative). The cross-contamination was discharged because of the saliva samples being collected prior to the endoscopic procedure and collection of gastric biopsies.

The phylogenetic analysis failed to determine the species of the *Helicobacter* detected in the individual samples. However, based on the closeness of the samples of this study with strains of *Helicobacter* species such as *H. heilmannii s.s*, *H. salomonis*, *H. felis*, and *H. bizzozeronii*, we can infer similarity between our samples with these NHPH species. These species of *Helicobacter* are important as they are considered to share close evolutionary relationships and genomic similarities with *H. pylori* [38–40]. This might explain the capacity of these NHPH to readily adapt to the gastric environment of humans and to induce gastric diseases, highlighting the zoonotic nature of these bacterial species [39]. The phylogenetic analysis of saliva samples was only possible to dog number 24, and the other samples were not performed due to a weak fluorescence signal.

Due to *Helicobacter* infection as an important risk factor, currently in Brazil the high mortality rate associated with gastric cancer, it was the fourth-largest cause of death due to neoplasms for men, and the sixth among women [41] and the prevalence and mortality rates due to peptic ulcers have increased as the population age increases [42]. However, the real percentage of these cases related to NHPH infection is unkown. Notably, as the human population increases their contact with domestic animals, a directly proportional increase in NHPH infected people could result relative to the socioeconomic underdevelopment of the country they inhabit [14]. Furthermore, saliva and faeces of dogs with NHPH may be potential sources of infection for human populations [9, 11]. Thus, this is what motivated the authors to initiate and develop this study, as we were faced with a growing canine population while poor sanitary conditions are still a reality in our country. Due to the zoonotic importance of NHPH bacteria, future cohort studies evaluating the prevalence of *Helicobacter* spp. in the Brazilian human population that could be directly associated with their pets would be extremely relevant in order to elucidate whether NHPH is a concern to public health.

## 5. Conclusions

The results of the present study demonstrated a low occurrence of *Helicobacter* spp. in the saliva, and a high occurrence, predominance, and density of zoonotic importance in the stomach of domestic dogs of the central region of Rio Grande do Sul, Brazil. The saliva of the domestic dogs may be a transmission reservoir of *Helicobacter*, even in animals without bacterial colonisation. The *Helicobacter* gastric presence and gastric colonisation density failed to show an association with the gastric lesions pattern observed during histopathology.

## Figures and Tables

**Table 1:** Detection of *Helicobacter* spp., in different tests evaluated and gastric bacterial colonisation density scores.

Animal no.	Chronic vomiting	Saliva		Gastric mucosa		
		16S rRNA PCR		Score cytology		RUT
		assay		Body	Antrum	
1	P	+	−	−	−	N/P
2	P	−	−	−	−	N/P
3	P	+	+	++	++	+
4	P	−	−	−	−	−
5	P	+	+	+	++	+
6	P	−	−	++	+	+
7	P	−	+	++	++	+
8	P	−	+	++	+	+
9	A	−	+	+	+++	+
10	A	−	+	++	+	+
11	A	−	+	+++	++	+
12	A	−	+	++	N/P	+
13	A	−	−	+	++	+
14	A	−	+	++	+++	+
15	A	−	+	+	N/P	+
16	P	−	+	++	+	+
17	A	−	+	++	+	+
18	A	−	−	+	N/P	+
19	A	−	−	++	++	+
20	P	−	+	+	+	+
21	P	−	+	+	+	+
22	A	+	−	++	+++	+
23	A	−	+	+	+	+
24	A	+	+	+	+	+
25	A	+	+	+++	+++	+
26	A	−	+	+++	N/P	+
27	P	+	+	+	+	+
28	A	−	+	++	+++	+
29	A	+	+	++	+	+
30	A	−	+	++	++	+
31	A	−	+	++	++	+
32	A	−	+	++	+++	+
33	A	−	+	+	++	+
34	A	+	−	+	−	−
35	A	−	+	+++	++	+
	Total	9/35	26/35	32/35	27/35	31/35

P: present; A: absent; RUT: Rapid Urease Test; N/P: not performed; −: negative; +: positive and mild score; ++: moderate score; +++: severe score.

**Table 2:** Findings of histologic gastric lesion on dogs colonised and noncolonised with *Helicobacter* in the stomach (mean of scores in both gastric zones).

Histologic gastric lesion [25]	Total (*n* = 35)	Colonised (*n* = 32)	Noncolonised (*n* = 3)
Mild gastritis	21	20	1
Moderate gastritis	1	1	0
Fibrosis and mucosal atrophy	3	2	1
Lymphocytic-plasmacytic infiltrate	18	17	1
Neutrophilic infiltrate	9	8	1
Eosinophilic infiltrate	9	8	1
Gastric lymphofollicular hyperplasia	3	3	0
Intraepithelial lymphocytes	4	4	0

**Table 3:** Case by case of frequency of the gastric bacterial colonisation density, presence of 16rRNA *Helicobacter* genus on saliva and stomach, and histologic findings with degrees of WSAVA classification.

Animal (no.) and history	16S rRNA PCR assay	Gastric bacterial colonisation density score	Histologic gastric lesion and degree (WSAVA classification)
1S	Saliva	Body: no bacteria Antrum: no bacteria	Body: normal Antrum: normal
2S	Negative	Body: no bacteria Antrum: no bacteria	Body: mild, lymphocytic-plasmacytic, eosinophilic and neutrophilic infiltrate Antrum: mild, neutrophilic infiltrate and fibrosis and mucosal atrophy
3S	Saliva, stomach	Body: moderate Antrum: moderate	Body: normal Antrum: normal
4S	Negative	Body: no bacteria Antrum: no bacteria	Body: normal Antrum: normal
5S	Saliva, stomach	Body: mild Antrum: moderate	Body: normal Antrum: normal
6S	Negative	Body: moderate Antrum: mild	Body: severe, lymphofollicular hyperplasia; mild, lymphocytic-plasmacytic and neutrophilic infiltrate Antrum: mild, lymphocytic-plasmacytic infiltrate
7S	Stomach	Body: moderate Antrum: moderate	Body: moderate, lymphofollicular hyperplasia; mild, fibrosis and mucosal atrophy, lymphocytic-plasmacytic infiltrate and intraepithelial lymphocytes Antrum: normal
8S	Stomach	Body: moderate Antrum: mild	Body: normal Antrum: NP ^*∗*^
9A	Stomach	Body: mild Antrum: severe	Body: normal Antrum: NP ^*∗*^
10A	Stomach	Body: moderate Antrum: mild	Body: mild, lymphocytic-plasmacytic infiltrate Antrum: NP ^*∗*^
11A	Stomach	Body: severe Antrum: moderate	Body: mild, lymphocytic-plasmacytic, neutrophilic and eosinophilic infiltrate and lymphofollicular hyperplasia Antrum: mild, lymphocytic-plasmacytic infiltrate
12A	Stomach	Body: mild Antrum: N/P	Body: mild, lymphocytic-plasmacytic infiltrate Antrum: NP
13A	Negative	Body: mild Antrum: moderate	Body: mild, lymphocytic-plasmacytic and neutrophilic infiltrate Antrum: mild, lymphocytic-plasmacytic and neutrophilic infiltrate
14A	Stomach	Body: moderate Antrum: severe	Body: NP ^*∗*^ Antrum: mild, lymphocytic-plasmacytic and neutrophilic infiltrate and intraepithelial lymphocytes
15A	Stomach	Body: mild Antrum: N/P	Body: mild, lymphocytic-plasmacytic, neutrophilic and eosinophilic infiltrate Antrum: NP
16A	Stomach	Body: moderate Antrum: mild	Body: normal Antrum: mild, lymphocytic-plasmacytic infiltrate
17A	Stomach	Body: moderate Antrum: mild	Body: mild, lymphocytic-plasmacytic and neutrophilic infiltrate Antrum: NP ^*∗*^
18A	Negative	Body: mild Antrum: N/P	Body: mild, lymphocytic-plasmacytic infiltrate Antrum: N/P
19A	Negative	Body: moderate Antrum: moderate	Body: normal Antrum: normal
20S	Stomach	Body: mild Antrum: mild	Body: normal Antrum: mild, lymphocytic-plasmacytic and eosinophilic infiltrate
21S	Stomach	Body: mild Antrum: mild	Body: normal Antrum: NP ^*∗*^
22A	Saliva	Body: moderate Antrum: severe	Body: normal Antrum: normal
23A	Stomach	Body: mild Antrum: mild	Body: mild, eosinophilic infiltrate Antrum: normal
24A	Saliva, stomach	Body: mild Antrum: mild	Body: normal Antrum: normal
25A	Saliva, stomach	Body: severe Antrum: severe	Body: normal Antrum: NP ^*∗*^
26A	Stomach	Body: severe Antrum: N/P	Body: mild, lymphocytic-plasmacytic infiltrate Antrum: NP
27S	Saliva, stomach	Body: mild Antrum: mild	Body: normal Antrum: NP ^*∗*^
28A	Stomach	Body: moderate Antrum: severe	Body: normal Antrum: mild, lymphocytic-plasmacytic, neutrophilic and eosinophilic infiltrate
29A	Saliva, stomach	Body: moderate Antrum: mild	Body: normal Antrum: mild, intraepithelial lymphocytes
30A	Stomach	Body: moderate Antrum: moderate	Body: mild, lymphocytic-plasmacytic and eosinophilic infiltrate and intraepithelial lymphocytes normal Antrum: mild, lymphocytic-plasmacytic and eosinophilic infiltrate
31A	Stomach	Body: moderate Antrum: moderate	Body: normal Antrum: mild, lymphocytic-plasmacytic infiltrate
32A	Stomach	Body: moderate Antrum: severe	Body: mild, lymphocytic-plasmacytic infiltrate Antrum: mild, neutrophilic infiltrate
33A	Stomach	Body: mild Antrum: moderate	Body: normal Antrum: mild, eosinophilic infiltrate
34A	Saliva	Body: mild Antrum: no bacteria	Body: mild, eosinophilic infiltrate Antrum: mild, eosinophilic infiltrate
35A	Stomach	Body: severe Antrum: moderate	Body: normal Antrum: normal

NP: not performed;  ^*∗*^: unviable sample; A: asymptomatic (healthy); S: symptomatic (chronic vomiting).

## Data Availability

The occurrence data of Helicobacters used to support the findings of this study are included within the article. The sequence data used to support findings of this article were deposited in GenBank; the accession numbers are Sample 3B: Genbank accession MN966439/Sample 5B: Genbank accession MN966440/Sample 7B: Genbank accession MN966441/Sample 8B: Genbank accession MN966442/Sample 9B: Genbank accession MN966443/Sample 10B: Genbank accession MN966444/Sample 11B: Genbank accession MN966445/Sample 12B: Genbank accession MN966446/Sample 15B: Genbank accession MN966447/Sample 16B: Genbank accession MN966448/Sample 17B: Genbank accession MN966449/Sample 20B: Genbank accession MN966450/Sample 21B: Genbank accession MN966451/Sample 23B: Genbank accession MN966452/Sample 24B: Genbank accession MN966453/Sample 24S: Genbank accession MN966454/Sample 25B: Genbank accession MN966455/Sample 26B: Genbank accession MN966456/Sample 27B: Genbank accession MN966457/Sample 28B: Genbank accession MN966458/Sample 29B: Genbank accession MN966459/Sample 30B: Genbank accession MN966460/Sample 31B: Genbank accession MN966461/Sample 32B: Genbank accession MN966462/Sample 33B: Genbank accession MN966463/Sample 35B: Genbank accession MN966464.

## Abbreviations

NHPH: Non-*H. pylori Helicobacter* species.

## Additional Points

*Limitations of This Study*. Unfortunately, the limitation in this study was the number of animals employed, which resulted in the limited duration of the study (14 months) and weak routine over this time. Samples from the antrum region were not possible to be collected due to difficult endoscopic access of the pylorus in four animals, and for histopathological analysis, some samples were not viable due to the insufficient amount of tissue or absence of the sample.
